# Supplementary material for: ECOD domain classification of 48 whole proteomes from AlphaFold Structure Database using DPAM2
Source: PLoS Comput Biol. 2024 Feb 28;20(2):e1011586. doi: 10.1371/journal.pcbi.1011586 (PMC10927120; doi:10.1371/journal.pcbi.1011586)
Supplement: S1 Table — (DOCX) [file pcbi.1011586.s001.docx]

**S1 Table. 48 Proteomes with DOL, identifiers, ordering, and % assigned.**

| AF2 name^1^ | Short name | DoL^2^ | Proteins | % well assigned^3^ | % classified | Taxonomic order^4^ |
| --- | --- | --- | --- | --- | --- | --- |
| 9euro1 | Cladc | Eukaryote | 11170 | 59% | 97% | 5 |
| 9euro2 | Fonpe | Eukaryote | 12509 | 64% | 97% | 6 |
| 9noca1 | Nocbr | Bacteria | 8372 | 83% | 98% | 37 |
| 9pezi1 | Madmy | Eukaryote | 9561 | 60% | 97% | 1 |
| ajecg | Ajecg | Eukaryote | 9199 | 55% | 97% | 4 |
| arath | Arath | Eukaryote | 27434 | 64% | 100% | 31 |
| bruma | Bruma | Eukaryote | 8743 | 56% | 97% | 24 |
| caeel | Caeel | Eukaryote | 19694 | 61% | 100% | 26 |
| camje | Camje | Bacteria | 1620 | 85% | 98% | 35 |
| canal | Canal | Eukaryote | 5974 | 63% | 100% | 9 |
| danre | Danre | Eukaryote | 24664 | 60% | 100% | 17 |
| dicdi | Dicdi | Eukaryote | 12622 | 56% | 100% | 10 |
| drame | Drame | Eukaryote | 10834 | 61% | 97% | 21 |
| drome | Drome | Eukaryote | 13458 | 54% | 100% | 19 |
| ecoli | Ecoli | Bacteria | 4363 | 86% | 100% | 46 |
| entfc | Entfc | Bacteria | 2823 | 84% | 98% | 32 |
| haein | Haein | Bacteria | 1662 | 87% | 98% | 47 |
| helpy | Helpy | Bacteria | 1538 | 81% | 98% | 36 |
| homsa | Human | Eukaryote | 23391 | 57% | 98% | 18 |
| kleph | Kleph | Bacteria | 5727 | 84% | 98% | 43 |
| leiin | Leiin | Eukaryote | 7924 | 48% | 97% | 11 |
| maize | Maize | Eukaryote | 39299 | 55% | 100% | 28 |
| metja | Metja | Archaea | 1773 | 84% | 100% | 48 |
| mouse | Mouse | Eukaryote | 21615 | 60% | 100% | 16 |
| mycle | Mycle | Bacteria | 1602 | 68% | 98% | 39 |
| myctu | Myctu | Bacteria | 3988 | 79% | 98% | 40 |
| mycul | Mycul | Bacteria | 9033 | 61% | 96% | 38 |
| neig1 | Neig1 | Bacteria | 2106 | 80% | 98% | 41 |
| oncvo | Oncvo | Eukaryote | 12047 | 57% | 97% | 23 |
| orsyj | Orsyj | Eukaryote | 43649 | 53% | 100% | 29 |
| parba | Parba | Eukaryote | 8794 | 55% | 97% | 3 |
| plaf7 | Plaf7 | Eukaryote | 5187 | 41% | 98% | 14 |
| pseae | Pseae | Bacteria | 5556 | 84% | 98% | 42 |
| rat | Rat | Eukaryote | 21272 | 60% | 100% | 15 |
| salty | Salty | Bacteria | 4526 | 85% | 98% | 45 |
| schma | Schma | Eukaryote | 13865 | 49% | 98% | 27 |
| schpo | Schpo | Eukaryote | 5128 | 68% | 100% | 7 |
| shids | Shids | Bacteria | 3893 | 84% | 97% | 44 |
| soybn | Soybn | Eukaryote | 55799 | 61% | 100% | 30 |
| spos1 | Spos1 | Eukaryote | 8652 | 57% | 97% | 2 |
| staa8 | Staa8 | Bacteria | 5776 | 83% | 98% | 34 |
| strer | Strer | Eukaryote | 12613 | 61% | 97% | 25 |
| strr6 | Strr6 | Bacteria | 2030 | 85% | 98% | 33 |
| tritr | Tritr | Eukaryote | 9564 | 64% | 98% | 20 |
| tryb2 | Tryb2 | Eukaryote | 8491 | 57% | 97% | 13 |
| trycc | Trycc | Eukaryote | 19036 | 50% | 97% | 12 |
| wucba | Wucba | Eukaryote | 12721 | 54% | 97% | 22 |
| yeast | Yeast | Eukaryote | 6040 | 63% | 100% | 8 |

**^1^**AF2 name largely coincides with UniProt proteome “mnemonic” names with some exceptions. Non-unique mnemonic names are modified (e.g. “9euro”) to be unique by AF2DB.

^2^Domain of life: Bacterial (B), Eukarya (E), or Archaea (A)

^3^% of residues within proteome that are within a “well-assigned” domain

^4^Taxonomic order determined by PHYLIP tree of NCBI taxonomy identifiers.
