# Supplementary material for: ECOD domain classification of 48 whole proteomes from AlphaFold Structure Database using DPAM2
Source: PLoS Comput Biol. 2024 Feb 28;20(2):e1011586. doi: 10.1371/journal.pcbi.1011586 (PMC10927120; doi:10.1371/journal.pcbi.1011586)
Supplement: S1 Fig — The increase in domains from the most used methods in the PDB. The increase in structures determined by electron microscopy does lead to a sizeable increase in (redundant) total domains as well as representative domain structures. (DOCX) [file pcbi.1011586.s002.docx]

**S1 Fig. Total and representative domains in ECOD from 2016 (v45) and 2022(v285) by experimental method.** The increase in domains from the most used methods in the PDB. The increase in structures determined by electron microscopy does lead to a sizeable increase in (redundant) total domains as well as representative domain structures.
