# Supplementary material for: ECOD domain classification of 48 whole proteomes from AlphaFold Structure Database using DPAM2
Source: PLoS Comput Biol. 2024 Feb 28;20(2):e1011586. doi: 10.1371/journal.pcbi.1011586 (PMC10927120; doi:10.1371/journal.pcbi.1011586)
Supplement: S2 Fig — The methods with the most sizeable increase between 2016 and 2022 were X-ray crystallography and electron microscopy. Electron microscopy structures commonly have many proteins (chains) as reflected in this data. (DOCX) [file pcbi.1011586.s003.docx]

­­

**S2 Fig. PDB depositions and chains classified by ECOD in 2016(v45) and 2022(v285).** The methods with the most sizeable increase between 2016 and 2022 were X-ray crystallography and electron microscopy. Electron microscopy structures commonly have many proteins (chains) as reflected in this data.
