# Supplementary material for: ECOD domain classification of 48 whole proteomes from AlphaFold Structure Database using DPAM2
Source: PLoS Comput Biol. 2024 Feb 28;20(2):e1011586. doi: 10.1371/journal.pcbi.1011586 (PMC10927120; doi:10.1371/journal.pcbi.1011586)
Supplement: S3 Fig — Domains of life determined by PDB metadata associated with NCBI Taxonomy Ids. Archaea (A), Eukarya (E), Bacteria (B), and Virus (V) along with Other (O) or Unknown (U) NCBI taxonomy categories. PDB metadata associated with obsolete PDB or NCBI taxonomy ids resolve to (Unk). (DOCX) [file pcbi.1011586.s004.docx]

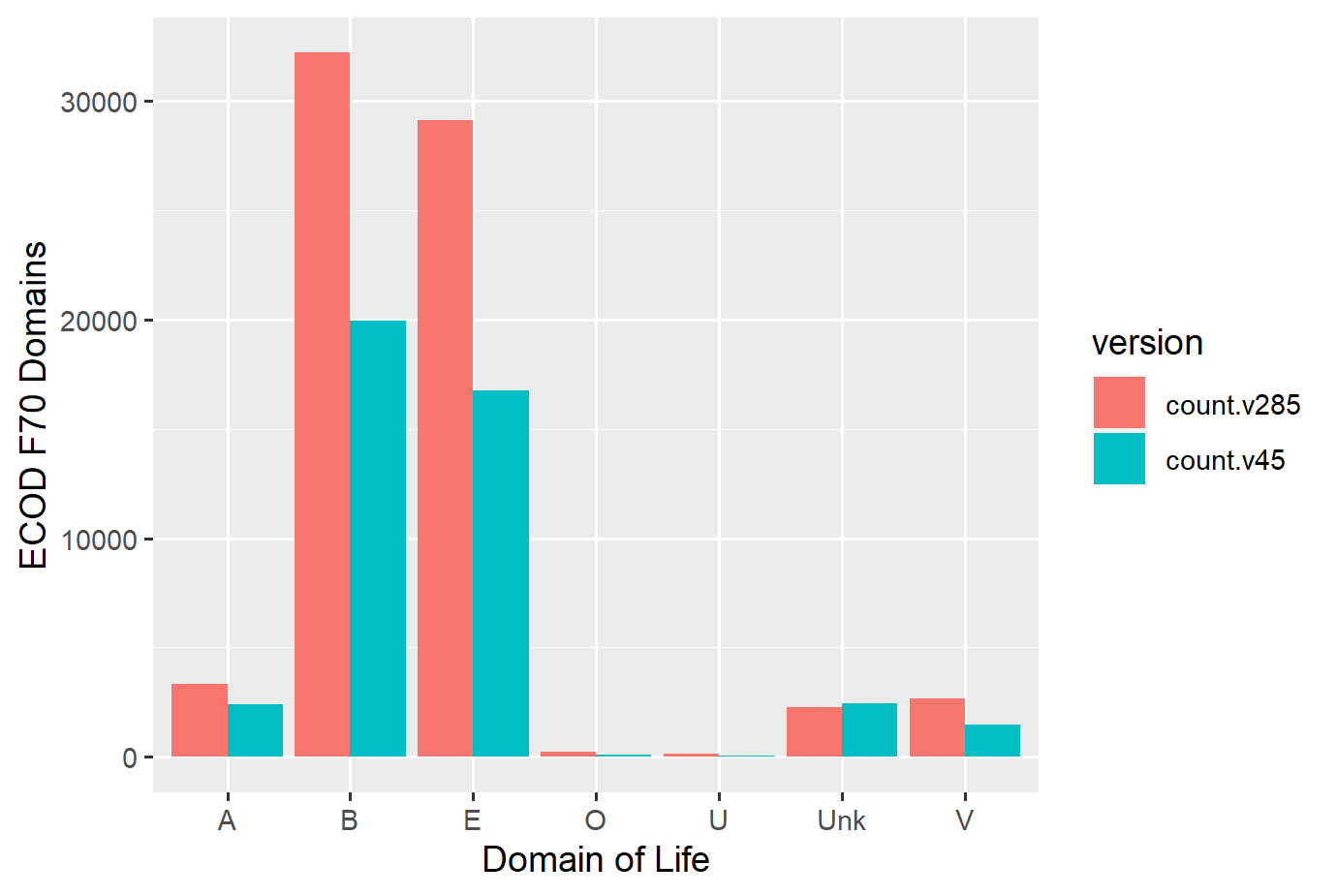


**S3 Fig.** **Domains of Life recorded in ECOD 2016(v45) and 2022(v285).** Domains of life determined by PDB metadata associated with NCBI Taxonomy Ids. Archaea (A), Eukarya (E), Bacteria (B), and Virus (V) along with Other (O) or Unknown (U) NCBI taxonomy categories. PDB metadata associated with obsolete PDB or NCBI taxonomy ids resolve to (Unk).
