# Supplementary material for: ECOD domain classification of 48 whole proteomes from AlphaFold Structure Database using DPAM2
Source: PLoS Comput Biol. 2024 Feb 28;20(2):e1011586. doi: 10.1371/journal.pcbi.1011586 (PMC10927120; doi:10.1371/journal.pcbi.1011586)
Supplement: Attachment — Submitted filename: Response to Reviewers.docx [file pcbi.1011586.s005.docx]

We appreciate the reviewers' comments, they came from a place of understanding and made us think about several issues in more depth. We apologize for any confusion from the state of the ECOD website(s) at the time of submission, we hope it is more clear in its current state. We hope you find the following response acceptable for publication.

Cheers!

Schaeffer et al.

*Reviewer #1: In this work, Schaeffer et al. describe their efforts on the classification of known ECOD domains onto the proteomes of 48 species across the tree of life, now possible due to the availability of structural models in the AlphaFold database. This is a timely work, as it allows us to infer about the distribution of known domains and folds in Nature, which is not accurately depicted by the Protein Databank. The paper is well written, and the ideas are well conveyed, but I have some major and minor issues. Please see below:*

Major:
*- I am disappointed to not see a discussion on novelties or global but non-assigned predicted domains. The authors write that 66% of residues could be assigned a domain, whereas 4% couldn't but don't discuss these further. What are their properties? are they mostly repetitive, helical, stranded, small, large, etc? Could they give origin to new X-groups, for example?*

We added a short section on potentially novel domains among the unassigned DPAM domains. These domains have a weak tendency to be all-alpha or somewhat unstructured, but come from all architectures. There are many cases of evolutionary arms race proteins(i.e. toxin anti-toxin systems), some boundary cases (i.e. weak homology beyond our confidence cutoff that require manual examination), and some assignment errors (i.e. arising from inconsistency between ECOD and DPAM’s implicit domain definition). They could give origin to new groups (both new X-groups and new H-groups in existing X-groups). We present an example of one of these domains and how it would be classified upon integration of these domains into a full ECOD classification. The full classification of these unclassified domains into new groups (and more generally a merged classification of experimental and predicted structures) is the scope of a forthcoming draft, but we here discuss an example and summary statistics of the group from this 48 proteomes set.

*- the authors mention in the abstract that they demonstrate that eukaryotic proteins are more repeated than bacterial ones, but that is not obvious from the main text. A figure highlighting the different tendencies for domain compositions between bacteria and eukaryota would allow to a better understanding of the different tendencies between the two different groups and would make the discussion stronger.*

Figure 3C breaks out the most populated H-groups by organism (wherein the organisms are separated by domain of life) including those groups that are dominated by internal repeitition (e.g. leucine-rich repeats, armadillo repeats). We have added some additional explanatory text to the main text as well as added eukarya, bacteria, archaea breakdowns (as in figure 1).

*- how are these domain annotations found in ecod? it is not clear from the manuscript neither the ECOD webpage. I would expect that querying by uniprot ac would allow for finding the domains assigned to them, for example, but that does not seem the case.*

The AF2 classifications are currently held separate from the ECOD PDB classifications. Integration of domains from predicted models and experimental structures are in the scope of a future work. Explicit web addresses for the AF2 classification have been included in the manuscript, repository, and main ECOD webpage to make this more clear. From the AF2 webpage, domains can be searched using UniProt accession.

*Minor:

- figure 1: the xticks are not easy to read because they are cramped into each other and also are not full species names*

We resized the elements of Figure 1. The abbreviations in Fig 1 correspond to the reference of species names and their short names in the supplementary materials. Briefly, the AFDB names loosely correspond to UniProt mnemonic names with some allowance for the non-uniqueness among those names (e.g., 9euro, 9euro1, and 9euro2). We maintain that the use of these abbreviations is a compromise between legibility and linkage to the base AFDB data. We also modified Fig 3D to reflect these same species abbreviations.

*- figure 2 legend: I think the labels of the figures in the legend text is shifted. B) appears twice. Also, it says "The HTH domain of this protein (purple) is shown in the context of other detected domains" but this is difficult to grasp in the figure. the three structure panels would be easier to interpret if the different domains were labelled. same in figure 3.*

We have corrected the error in panel labels. We have also added domain labels to more clearly identify domains in structure panels in Figures 2 and 3.

*- figure 3: in panel D, it is not obvious the separation between bacteria and eukaryotes. highlighting them as in figure 1 would facilitate the interpretation of the figure.*

We have incorporated a similar separation between the domains of life in Figure 3C(previously D).

*-p 8 line 134: Fig. 1 mycle, you mean "myucl"?*

We have corrected the error.

*- p19 line 390: the citations are wrongly placed and misleading. they are for pymol, R and ggplot and not for excel.*

We have reorganized the citations in these locations for clarity.

*- p 19, line 408: the authors write "lower resolution" but i guess what they mean is "higher resolution", ie. lower value*

This error has been corrected.

*Reviewer #2: The manuscript “ECOD domain classification of 48 whole proteomes from AlphaFold Structure Database using DPAM” by RD Schaeffer et al (PCOMPBIOL-D-23-01629) describes a prospective extension of the evolutionary classification of protein domains (info on experimental structures in the ECOD database (*[*http://prodata.swmed.edu/ecod/*](http://prodata.swmed.edu/ecod/)*) towards models of individual protein structures produced by AlphaFold2 which are held in the AlphaFold Protein Structure Database (*[*https://alphafold.ebi.ac.uk/*](https://alphafold.ebi.ac.uk/)*). The results described in the manuscript comprise models from 48 proteomes (excluding human described in the previous work from the same lab). The authors found that ~75% proteins in the considered proteomes have high-confidence domains similar/homoogous to the domains previously identified for the experimental structures in the earlier ECOD release, maintained by the same lab. Additionally, ~ 5% (from Fig 1 in the manuscript) of proteins have domains that do not have any similarity/homology detected by the methods used. Authors also provided comparative analysis of new domains as well as of the annotated domains in different kingdoms of life. In general, subject of the manuscript is extremely relevant for the field of structural molecular biology and worth publishing in PLoS Computational Biology. Below are some comments/suggestions that can, in my opinion, improve quality of the manuscript.*

*1. Title of the manuscript should reflect the fact that DPAM used in this study differ from earlier DPAM used to annotate models of human proteome.*

We have altered the title to clarify that the version of DPAM used in this study differs from the human proteome version.

*2. Number of domains provided on line 106 (p. 6 of the manuscript), 898 379, contradicts number 746 349 given in the abstract. The latter corresponds to the number of entries in the file af2_all_dpam_v5_domains.txt, available at Zenodo repository. Also, those numbers are surprisingly close to the numbers from previously released domain annotation for the experimental structures (line 108 on p.6). Authors should clarify and comment on that situation.*

This was indeed a typo. 746,349 is the correct number of well-assigned domains from predicted models. 898,379 is the correct number of domains (manual and automatic) from PDB structures (at the time of writing). We have corrected the error.

*3. The data are actually not available from the main ECOD webpage and thus authors either should remove the mentioning of ECOD on line 109, p.7, or add corresponding links to the ECOD webpage. Also, it would be highly desirable if ECOD users could search for specific domains in both experimental and model AF2 structures.*

The situation was indeed confusing on the webpage. We have added a URL for the AF2 ECOD website in the manuscript, as well as additional navigation links to the main ECOD website for the AF2 website. We agree that the integration of the experimental structure and predicted data would be a useful tool but it is out of scope for this work. The technical and interface challenges regarding this integration (between the PDB, AF2, DPAM v1 Human, Vibrio RIMD, and one further work in preparation) will be the subject of future database/website-focused work. We think it is useful for both reproducibility and review to have a separate site/repository simply focused on the 48 proteome classification.

*4. In figure 1, color palette used in the figure disagree with the description of colors in the figure caption. For instance, assigned domains are in blue in the figure, but claimed to be gray in the caption. This should be corrected.*

The submitted color palette reflected a prior figure version, it has been corrected.

*5. Ordering by taxonomy ID mentioned on lines 132 and 133 on p.8 should be explained more in details.*

We have added some detail (referenced the NCBI Taxonomy CommonTree tool) as well as added a citation to the tool. This is not a phylogenetic analysis as much as a way to group proteomes by their commonly accepted taxonomy in a broad way.

*6. Short name ‘Mycle’ on line 134 should be actually ‘Mycul’.*

We have corrected the error

*7. Percentages given on line 155, p.8 are misleading as they refer to the percentage inside only one fragment of inner pie. That should be explained better. Additionally, Fig 2A would convey the information better if fractions/percentages will be added directly to the chart with explanation that fraction in the inner pie are with respect to the entire circle and fractions in the outer pie corresponds to one of the segments in the inner pie. Fig 2D would also benefit, if all non-magenta domains will be blurred. Also, lines 182 – 186 and 188 – 190 from the caption of this figure belong to the discussion in the main text rather then to the figure caption, which should, in my opinion, contain only factual info related to the displayed picture(s).*

We agree this data needs clarification. We removed the single “fraction of class” percentage, these data are incorporated in the supplementary materials and are probably of less interest to the average reader. This left only fraction of all percentages explicitly in the main text. The fraction of all percentages have been added to the inner ring of Fig 2A. For figure 2D, we have adopted R1’s suggestion for explicit domain labels for domains of interest, we hope that will address this point as well. Lines 182-186,188-190 have been removed to the main text.

*8. Panel C in fig 3 is redundant as all info is in panels A and B. Also, Reference to panel D is missing in the main text.*

We removed panel C as the small amount of novel data it contained didn’t warrant inclusion (highly populated H-groups that are unique to eukarya or bacteria) and those data are better represented in panel C. We added a reference to panel D (now C) in the text.

*9. Fig 4B does not display relative population (as it is claimed in the main text and in the caption), thus it is difficult to judge whether it supports statement in the text or not. Also, sentence on line 279, p 14, belongs to the main text rather than figure caption.*

Figure 4B compares domain populations (F70) between “old” and “new” ECOD directly, rather than by relative population. We have altered the text to correct this typo.

*10. Line 286, p.14. Should it be “under-sampled by experimental methods”?*

We have clarified this sentence.

*11. Does “low confidence domains” refer to low conf region or unassigned domain in Fig 1?*

It referred to unassigned domains, which is confusing. We have clarified this sentence by changing “low confidence domains” to “unassigned domains”. We have also added a short description of how the Fig1 “low confidence regions” and “flexible regions” were determined from regions that DPAM could not classify.

*Reviewer #3: The author used ECOD domain classification to annotate 48 proteomes from AlphaFold Structure Database. Overall, the paper has some significance, but there are still some important issues that need to be addressed.

1. The author concludes that eukaryotes contain a higher proportion of disordered regions and unassigned domains compared to bacteria. However, these findings seem to have been previously proposed, as referenced in the papers by Bordin et al. [1] and Porta-Pardo et al [2]. Specifically, Bordin et al. conducted a structural analysis of 21 protein complexes predicted by AlphaFold, and the difference appears to lie in the use of different annotation methods (CATH). Nevertheless, both studies reached similar conclusions. Therefore, I believe the author should emphasize their own strengths in light of these existing findings.
reference:
[1] Bordin, Nicola, et al. "AlphaFold2 reveals commonalities and novelties in protein structure space for 21 model organisms." Communications Biology 6.1 (2023): 160.
[2] Porta-Pardo, Eduard, et al. "The structural coverage of the human proteome before and after AlphaFold." PLOS Computational Biology 18.1 (2022): e1009818.*

We have added additional context to the introduction and discussion to reflect these previous works. Specifically, both the Bordin et al. and this work find a similar fraction of models from predicted domains that can be assigned to known protein homologous groups, and they also observed a drop in assignment in eukaryotic organisms. The CATH work features an extensive manual curation of human proteins, in our case ECOD human protein curation was confined to a previous work. Nevertheless, we have added a short discussion of unassigned domains from these 48 proteomes.
 *2. Did the author consider the partial domains as discontinuous domains? Although they may be discontinuous in sequence, they are close in structural space.*

Discontinuous domains are explicitly allowed and designed to be defined by DPAM and ECOD. Partial domains are a small fraction of the defined domains and may belong to a small number of anomalous categories. Partial domains, containing strong signal but low coverage to reference domains, would commonly be domain fragments in experimental structures, usually a consequence of construct preparation. In predicted models, we need to look to other causes. Usually they are due to some sort of genome annotation error (perhaps a missing exon in eukaryotes), or a deteriorated and possible untranslated cryptic domain. Also, they can be due to inconsistencies in the reference. For example, a single member of a homodimeric transmembrane domain finding an unsplit duplication in the ECOD reference. It is possible that it could be due to a very long discontinuity, but as the MFS example shows, one of the strengths of the ECOD classification method is that it can find discontinuous domains even when the insertion is unstructured or unknown due to the closeness in strtuctural space

*3. In order to identify inherently disordered regions, tools like IUPred can be used. Relying solely on the evaluation metrics of AlphaFold is limited when it comes to recognizing disordered regions.*

DPAM does use independent measures of disorder, such as DISOPRED, in the partition and assignment steps of domain classification. Also, DSSP is used to determine the absence or presence of secondary structure in the predicted model. Nevertheless, care should be taken in discussing regions that appear unstructured in models, as it can be due to both technical (underprediction) as well as biological (intrinsic disorder) reasons, and these can be difficult to disentangle simply from self-reported quality measures.

*4. The references of the paper need to be carefully checked. For example, reference 20 should be 23, the reference of the alphafold on page seven, and the position of references 52-54.*

In this case we think it is useful to reference both the original PNAS Rives ESM-Fold paper (20) as well as the ESM-Fold2 model that was used to generate ESM-Atlas(23). We fixed the location of references 52-54
 *5. Authors should also carefully check that abbreviations are consistent throughout the paper. For example, AFDB and AF2DB.*

We have standardized this abbreviation to “AFDB”
